# Fertility Sparing Treatment of Endometrial Cancer with and without Initial Infiltration of Myometrium: A Single Center Experience

**DOI:** 10.3390/cancers12123571

**Published:** 2020-11-29

**Authors:** Paolo Casadio, Mariangela La Rosa, Andrea Alletto, Giulia Magnarelli, Alessandro Arena, Enrico Fontana, Matilde Fabbri, Kevin Giovannico, Agnese Virgilio, Diego Raimondo, Francesca Guasina, Roberto Paradisi, Renato Seracchioli

**Affiliations:** 1 Gynecology and Human Reproduction Physiopathology Unit, IRCCS Policlinico di Sant’Orsola, DIMEC, University of Bologna, 40138 Bologna, Italy; p.paolocasadio@gmail.com (P.C.); mariangela.lr89@gmail.com (M.L.R.); alessandro.arena6@unibo.it (A.A.); enricofontana15@gmail.com (E.F.); matilde.fabbri90@gmail.com (M.F.); kevin.giovannico@studio.unibo.it (K.G.); agnese.virgilio@studio.unibo.it (A.V.); die.raimondo@gmail.com (D.R.); roberto.paradisi@unibo.it (R.P.); renato.seracchioli@aosp.bo.it (R.S.); 2 Department of Gynecology and Obstetrics, Santa Chiara Regional Hospital, 38122 Trento, Italy; francesca.guasina@gmail.com

**Keywords:** endometrial cancer, fertility-sparing, hysteroscopy, hormonal therapy, progestogen

## Abstract

**Simple Summary:**

Endometrial cancer is the most common malignancy of the female genital tract, and in 14% of cases, is diagnosed in premenopausal women, While, the cancer appears in 5% in women of childbearing age. Preserving fertility in these women should be the goal of cancer practice. The aim of our study is to describe pregnancy outcomes of our center in women with G1 endometrial endometrioid cancer and atypical endometrial hyperplasia/endometrial intraepithelial neoplasm undergone conservative treatment. Moreover, for the first time, obstetric and oncological outcomes are described in a long follow up, including women with minimal myometrial infiltration.

**Abstract:**

Endometrial cancer (EC) is the fourth largest female cancer in Europe and North America. In 5% of cases, the diagnosis is made in women who wish to become pregnant. In our retrospective study, we reported our experience about fertility sparing treatment of G1 endometrioid endometrial cancer (G1 EEC) or atypical endometrial hyperplasia/endometrial intraepithelial neoplasm (AEH/EIN) in young women desiring pregnancy treated in our Center. Conservative treatment was based on operative hysteroscopy and hormone therapy with megestrol acetate (160 mg/die for 9 months). For the first time we included women with G1 EEC with minimal myometrial infiltration. The minimum follow-up period was two years and consisted of serial outpatient hysteroscopies with endometrial biopsies. Among the 36 women with G1 EEC we observed one case of disease persistence and four recurrences and four recurrences among the 46 women diagnosed with AEH/EIN. To date, 35 live births were obtained in both groups. Our results advance the hypothesis that conservative treatment can represent a safe and feasible alternative to propose to young women with desire for pregnancy. Further randomized and multicentric studies are needed to arrive at unambiguous and standardized guidelines on the surgical and medical treatment of young women with EEC or AEH/EIN.

## 1. Introduction

Endometrial cancer (EC) is the most common cancer of the female genital tract and the fourth most frequent after breast, lung, and colorectal cancers in Europe and North America [1]. It is the fifth most common cancer in women, who have a 1% cumulative risk of developing the disease within 75 years [2]. Although, the incidence of endometrial cancer is rising with increasing life, due to its common association with post-menopausal age, 14% of cases are diagnosed in premenopausal women and 5% of whom are under 40 [3,4]. In these young women, the majority of cases are endometrioid, focal, well-differentiated EC, limited to the endometrium or superficial myometrium which generally shows a good long-term prognosis [3,5].

These data highlight the importance of preserving fertility in these young women. The conservative management of early stage EC is generally well-accepted in young women wishing to preserve their fertility or in women who have severe surgical risk factors [6,7]. In fact, hysteroscopic resections accompanied by hormonal therapy for fertility-sparing treatment in young women with early-stage EC were reported in several studies [6,8,9], and it appears to be a reasonable opportunity in these cases with specific criteria. In particular, conservative management was proposed to women with grade 1 (G1) well-differentiated Endometrial Endometrioid Cancer (EEC), with no infiltration of the lymphovascular spaces and no evidence of myometrial invasion, and of metastatic disease or suspected adnexal mass [8].

On the other hand, in young women with G1 EEC with minimal myometrial invasion (infiltration of the myometrium less than 3 mm), who definitely refuse hysterectomy and wish to maintain their fertility, it is difficult to select a safe therapeutic alternative to traditional surgery. Our group recently reported a pilot study on the management of fertility-sparing in women with well-differentiated G1 EEC with minimal myometrial infiltration preventively treated with hysteroscopic resection and hormone therapy [10]. On the other hand, there are no definitive data on women selection criteria and an optimal therapeutic and follow-up managements.

In this study we report our experience on the fertility sparing treatment of G1 EEC and atypical endometrial hyperplasia/endometrial intraepithelial neoplasm (AEH/EIN) in young women wishing to become pregnant treated in our tertiary Center.

## 2. Results

**Figure 1:**
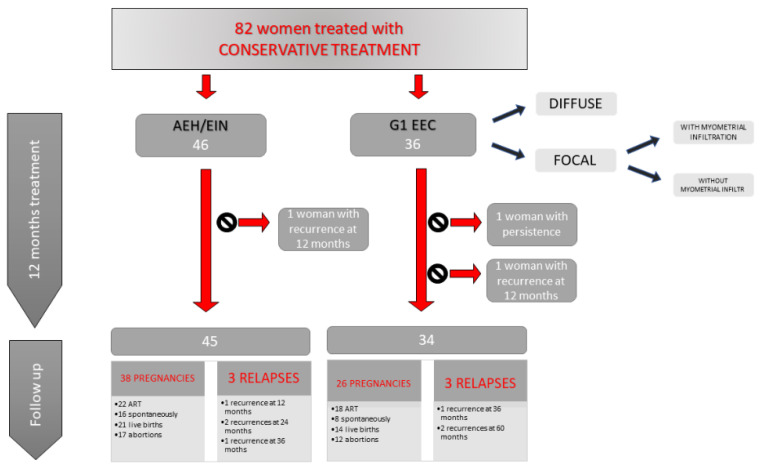
The figure shows our results in terms of persistence and relapse of disease and obtained pregnancies.

In the study 46 women with diagnosis of EEC and 55 women with diagnosis of AEH/EIN were included (Figure 1).

After the initial counselling ten (21.7%) women with G1 EEC and nine (16.3%) women with AEH/EIN refused conservative treatment and underwent total hysterectomy with bilateral salpingectomy, due to the risks associated with the proposed treatment, the long and strict follow-up period and because some of these had already exhausted their desire for pregnancy.

The baseline characteristics of the women studied are showed in Table 1.

Among the women with diagnosis of G1 EEC, two of them suffered from hypertension and type 1 diabetes was reported in one case.

Among the women with diagnosis of AEH/EIN, we found three women with hypertension and two women with type 2 diabetes.

### 2.1. Women with G1 EEC

Of the 36 women with G1 EEC, diagnosed at operative hysteroscopy and undergoing conservative treatment, 8/36 (22.2%) had diffuse intracavitary disease without myometrial infiltration and 28/36 (77.8%) had a focal development of the disease confirmed on histologic examination. In this second group, 2–3 mm myometrial infiltration was evidenced histologically in 5/28 cases (17.8%), despite the absence of lymphovascular invasion and the presence of free resection margins. Random endometrial samples were negative in all women. These women with myometrial invasion were informed about the main risks of disease persistence or recurrence, but they continue to refuse total hysterectomy and strongly wanted to continue conservative treatment.

During 9 months of megestrol acetate treatment 34/36 (94.4%), women achieved a complete remission of the pathology evaluated during outpatient hysteroscopy. In two cases in the group with intracavitary diffusion of the pathology, the lesion persisted at the first control. These women continued treatment with megestrol acetate and in one of them persistent disease was confirmed after six months. This patient underwent total hysterectomy and bilateral salpingectomy, and the histologic diagnosis was confirmed after surgery. The other woman with persistent disease was negative at the subsequent controls.

During follow-up, disease recurrence was observed in four women. In one case with myometrial infiltration, recurrence occurred as G1 EEC at three months after the end of hormonal treatment. Another woman without myometrial infiltration recurred as an EIN at 36 months after medical therapy. The last two cases occurred as AEH after 60 months of follow-up in women with myometrial invasion. All of these patients underwent total hysterectomy, and the histologic diagnosis was confirmed after surgery.

In relation to the reproductive outcomes of the thirty-four patients with complete remission at the first control after hormonal treatment and therefore authorized to seek pregnancy, twenty-one (61.7%) managed to obtain it for a total of twenty-six pregnancies. Eighteen (69.2%) of these were obtained with ART and resulted in eleven abortions and seven live births, while the other eight (30.8%) obtained spontaneously resulted in one abortion and seven live births. Our of the twenty-six pregnancies achieved in this group we obtained a total of twelve (46.2%) abortions and fourteen (53.8%) live births.

We performed total hysterectomy in three women with complete remission of the disease. In the first two cases, they exhausted their childbearing desire, while the third decided to undergo radical treatment due to her poor compliance. No evidence of oncological disease was shown on histologic examination in all cases.

### 2.2. Women with AEH/EIN

From the 46 women studied, we completed the remission of the disease in all cases during 9 months of megestrol acetate treatment.

During a median period of follow-up of 36 months (24–60) we assisted to disease recurrence in four cases. Two women relapsed as AEH/EIN to the control at three months after the end of hormonal treatment, and after 24 months, respectively, and two more relapsed as G1 EEC after 24 and 36 months, respectively. These women underwent total hysterectomy and histologic diagnosis was confirmed after surgery.

In this group, excluding the only one woman who recurred at the control of three months after progestin cessation, 45 women were authorized to seek pregnancy. Thirty-one (68.8%) became pregnant for a total of thirty-eight pregnancies. Twenty-two (57.8%) of these were obtained with ART, while sixteen spontaneously (42.2%). Among the pregnancies with ART, ten (45.5%) were live births and twelve (54.5%) resulted in abortions, while among spontaneous pregnancies, eleven (68.7%) were live births and five (31.3%) resulted in abortions.

From a total of thirty-eight pregnancies, we registered seventeen (44.7%) abortions and twenty-one (55.3%) live births.

We performed total hysterectomy in four women with complete remission of the disease after delivery at their request. No evidence of oncological disease was shown on histologic examination in all cases.

## 3. Discussion

EC occurs in women in premenopausal age in 14% of the cases and 5% are under 40 years. In recent decades we observed a change in childbearing age, due to an increasing number of women over the 35 years nulliparous and eager for offspring [11]. Therefore, often at the time of the diagnosis, women must preserve their fertility to complete their desire of pregnancy. The recommendations for fertility sparing treatment are based on the generally favorable prognosis of G1 minimally invasive EEC and pre-neoplastic lesions, as reported in several case series and case reports. Here, conservative treatment is usually offered to women under the 45 years with endometrium limited well-differentiated G1 EEC and no evidence of extra-uterine spread [6,9,12,13]. Moreover, the women must be highly motivated to maintain their reproductive function [9]. This strict women selection allows to highlight a good response rate to fertility sparing treatment and a low risk of recurrence or future extrauterine spread of the pathology. Furthermore, in case of persistence or recurrence of the disease, they can be treated with total hysterectomy without compromising the prognosis which seems to be excellent [14].

Office hysteroscopy is an accurate and less invasive method to evaluate several gynecologic disorders. A systematic review by Clark showed that the overall sensitivity and specificity of hysteroscopy for endometrial cancer were 86.4, and 99.2%, respectively [15]. Different techniques for endometrial biopsy such as blind-office procedures, dilation and curettage showed lower accuracies in diagnosis of G1 EEC and AEH/EIN compared to hysteroscopy and could cause intrauterine adhesions [16,17]. Office hysteroscopy allows a direct visualization of uterine cavity, in order to perform targeted biopsy and remove the lesion and to obtain endo-myometrial samples.

In our study, all women enrolled were aware of the risks of conservative surgery, the progression and recurrence of disease, and the need for a close follow-up. After a median of follow-up of 30 and 36 months in women with G1 EEC, and AEH/EIN, respectively, we observed disease persistence in two women at the first control and disease recurrence in eight.

Our hormone treatment consisted in megestrol acetate 160 mg per day for at least nine months. In the literature, there is no consensus about the best administration route for progestin therapy in case of early endometrial cancer. Several progestin therapy and several way of administrations have been used in fertility sparing treatment, but no one of them seems to be preferable [14,18].

The duration of the treatment period has not been clearly defined. The efficacy of conservative treatment in the international literature is reported to be between 3 and 12 months [6]. We believe that a treatment period of nine months could be effective, despite the fact that there are no clear data in the literature and it is still a matter of debate for fertility sparing procedures for endometrial cancer.

We observed an adequate response to conservative treatment in women with preneoplastic lesion (AEH/EIN), even when the entire uterine cavity was affected, with an initial complete remission in all cases and with a low number of recurrences in the follow-up. These outcomes are similar to those reported in the literature and show the efficacy of progestin therapy in superficial pre-neoplastic lesions [13,18]. Our study showed a complete remission at the first control in 94.4% of cases diagnosed with G1 EEC undergoing combined therapy and this finding agrees with those of other studies that indicated a response rate between 78% and 100% [6,7]. In our study, the cases of recurrence are slightly higher than those reported by Giampaolino et al. [6], likely because we carried out a longer follow-up and included women with minimal myometrial infiltration. Despite the good outcomes shown in our and other studies, the risk of recurrence cannot be overlooked in these women undergoing conservative treatment and therefore a strict follow-up is mandatory. Our proposal is based on tailored endometrial biopsies performed by outpatient hysteroscopy every three months for the first year and every six months for the next four years. Good compliance by women who must be immediately informed of the need for periodic controls is essential.

The main goal of the conservative treatment of EEC or AEH/EIN is pregnancy. Regarding reproductive outcomes, in our series 52 women achieved pregnancy in both groups. Although our data are not comparable with data reported by other Authors in terms of live birth rate, we report the data presented by Giampaolino et al. [6], Falcone et al. [9] and De Marzi et al. [19], who showed a live birth rate of 40%, 50% and 26%, respectively, considering EEC and EIN/AEH together. Peiretti et al. [20] reported in their review a pregnancy rate of 73.4%. Maybe due to our long period of follow-up, we obtained good results in terms of achievement of pregnancy and in our opinion this datum must be reported during counselling in women affected by endometrial cancer and scheduled for conservative treatment. We did not find substantial differences in terms of pregnancy outcomes between the two groups.

Nowadays case selection criteria, choice of surgical or medical treatment, follow-up strategies and the time to perform definitive surgical therapy are not uniform [9], and there is no consensus on the right time to perform a radical surgery in cases without cancer recurrence. Some authors recommend hysterectomy after pregnancy [21], but in our experience, prolonged follow-up seems to be safe in this group of women.

The strengths of our study, include (a) longer follow-up duration than that proposed by other authors [6], and (b) that for the first time we offer conservative treatment in cases with initial myometrial infiltration. In our experience, this treatment seems feasible and safe enough, even in women with myometrial infiltration, albeit the limitation due to the small number of women studied. Another important limitation of the study is its retrospective nature.

## 4. Material and Methods

The study was designed as a retrospective case series. We evaluated the obstetric and clinical outcomes of all women with G1 EEC or AEH/EIN and treated with fertility preservation treatment in our center between January 2010 and June 2019. The minimum follow-up period to enter in this series was 24 months after the end of progestin treatment. A protocol for collection of data for research purposes of women affected by endometrial cancer and submitted to fertility sparing treatment was approved by our local ethic committee with the specific code (3012020Sper/AOUBo). Moreover, due to the study’s retrospective design, a notification was performed, and the local Ethics Committee approved the collection of data for research purposes. All women provided signed informed consent during preoperative evaluation for unidentified data collection.

### 4.1. Subjects

**Figure 2:**
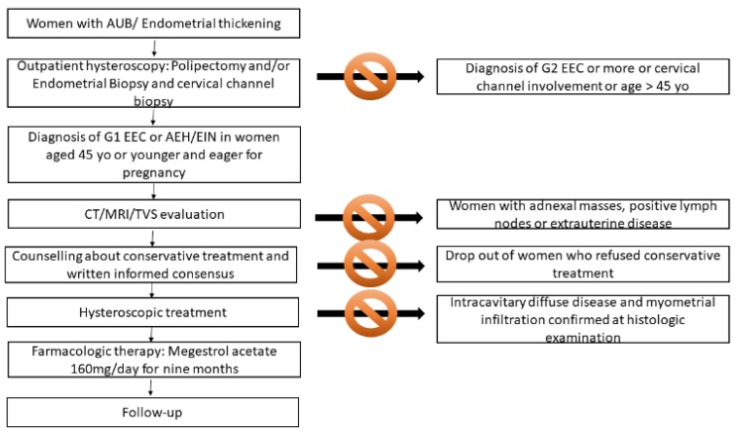
Flowchart of our diagnostic and therapeutic approach of women with G1 EEC and AEH/EIN candidate to fertility sparing treatment.

Women were sent to our out-patient hysteroscopy center for bleeding and abnormal endometrial thickness or suspected endometrial polyp after transvaginal sonography (TVS) examination. All the women underwent outpatient hysteroscopy performed by one experienced surgeon (P.C.), using a 5-mm hysteroscope with a 30° forward oblique lens (office hysteroscope; Karl Storz, Tuttlingen, Germany) and saline solution. During the hysteroscopy, polyps were removed and/or endometrial biopsies were performed in case of suspected areas of endometrial neoplasms. A biopsy of the cervical channel was systematically performed to rule out cervical involvement (Figure 2).

### 4.2. Inclusion and Exclusion Criteria and Counselling

In our Center, we offer fertility sparing treatment in women aged 45 years or less who wish to become pregnant with a diagnosis of G1 EEC or AEH/EIN.

After diagnosis, pre-conservative treatment work-up consisted of physical examination and TVS for AEH/EIN. Computed tomography (CT) and enhanced MRI were also added for G1 EEC to evaluate the presence of metastatic disease, pelvic or para-aortic lymph node involvement, suspicion of adnexal masses and evident myometrial invasion. If extra-uterine disease, clear myometrial infiltration or G1 EEC with diffuse intracavitary disease (a lesion that involve 25% or more of the uterine cavity) and suspected myometrial invasion were present, women were excluded from conservative treatment.

All women recruited strongly refused hysterectomy, so we offered them accurate counselling on the possibility of fertility-sparing treatment and the risks associated with non-standard treatment, after which they provided written informed consent to the conservative procedure. Women were aware of close follow up and the risk of hysterectomy in case of persistence or recurrence of the disease. They were also advised of the need of progestin therapy after surgical treatment, of the strict follow-up and that the goal of fertility sparing treatment was to achieve pregnancy.

### 4.3. Surgical Treatment

Operative hysteroscopy was performed by the same expert surgeon (P.C.) under general anesthesia; 10 mm cervical dilatation with Hegar dilators was accomplished and a 26-Fr resectoscope (Karl Storz, Tuttlingen, Germany) with a 0° lens was introduced. The uterus was distended with irrigation of a solution of 0.54% mannitol and 2.7% sorbitol urologic (Baxter Healthcare Corporation, Rome, Italy). Intrauterine pressure was automatically controlled (80–100 mm Hg) by an electronic irrigation and suction device (Hamou Endomat Irrigation Suction Pump, Karl Storz). In case of focal lesion, the endometrial lesion and a small portion (about 3–4 mm) of the underlying myometrium and the endometrium and myometrium surrounding the lesion were cautiously resected with a 5-mm cutting loop electrode and 100 watts of pure cutting output power, according to the technique of Mazzon et al. [8]. Additionally, multiple random endometrial biopsies were taken on each uterine wall. For women with diffuse intra-cavitary G1 EEC the hysteroscopic procedure consisted of multiple random endo-myometrial biopsies. All individual samples were sent in separate containers for histologic examination and were examined by the same experienced pathologist. On the other hand, women with diffuse intra-cavitary G1 EEC and myometrial infiltration confirmed at histologic examination were then excluded from conservative treatment. The presence of myometrial infiltration < 3 mm histologically evaluated in patients with G1 EEC focal lesion was not an exclusion criterion for conservative treatment.

### 4.4. Hormonal Treatment and Follow Up

**Figure 3:**
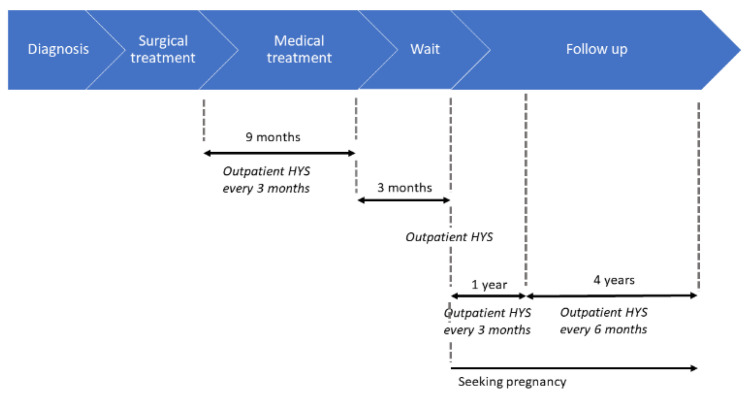
Timeline of the steps of the treatment and subsequent follow-up.

After the surgical procedure, the women were treated with progestin treatment (megestrol acetate 160 mg/day) for nine months (Figure 3). During progestin therapy women underwent outpatient hysteroscopy with endometrial biopsies every three months. Three months after progestin cessation, women underwent further outpatient hysteroscopy with multiple endometrial biopsies.

After a further three months from the last outpatient hysteroscopy and in case of negative biopsies, women were authorized to look for a pregnancy. They were also informed of the need to seek pregnancy using assisted reproductive technologies (ART) and underwent hysterosonosalpingography, partner spermiogram, antral follicular count by TVS and antimullerian hormone evaluation. Women who refused to seek pregnancy with ART were authorized to seek pregnancy spontaneously if the above tests were normal. Follow-up consisted of outpatient hysteroscopy with endometrial biopsies every three months for the first year and every six months for the next four years. In case of pregnancy, follow-up resumed after three months after delivery.

## 5. Conclusions

Fertility sparing treatment is playing an increasingly key role in the management of endometrial neoplastic disease in young women. Accurate advice on the risks and benefits of this treatment is recommended. Here, we show that conservative treatment seems to be safe and feasible, giving young women the chance to achieve a desired pregnancy. Unambiguous guidelines and prospective studies would be necessary to standardize surgical and medical therapy and treatment indications. On the other hand, the role of myometrial infiltration as an exclusion criterion should also be re-evaluated according to our outcomes that introduce the possibility of proposing this new minimally invasive treatment also to women with minimally infiltrating G1 EEC.

## Figures and Tables

**Table 1:** The baseline characteristics of the women with G1 EEC and AEH/EIN included in our study.

Characteristics of Women	G1 EEC	AEH/EIN
Total (*n*)	36	46
Age (mean ± standard deviation)	33.1 ± 4.77	32.2 ± 4.52
BMI (mean ± standard deviation)	29.1 ± 6.40	27.0 ± 4.61
Months of follow-up (median, range)	30 months (24–60)	36 months (24–60)
With myometrial infitration (<3 mm) (*n*)	5/36 (13.8%)	
Without myometrial infiltration (*n*) Diffuse intracavitary lesion Focal lesion	31/36 (86.2%) 8/31 (25.8%) 23/31 (74.2%)	
